# Meta-Analysis of Face-Validity Indicators in Automatically Detected Dog Sleep Spindles

**DOI:** 10.1007/s12021-026-09810-4

**Published:** 2026-08-15

**Authors:** Ivaylo Borislavov Iotchev, Anna Kis

**Affiliations:** HUNREN Research Center for Natural Sciences, Institute of Cognitive Neuroscience and Psychology, Budapest, 1117, 0.022 Hungary

**Keywords:** Sleep spindles, EEG, Polysomnography, Dogs, Automatic detection, Visual inspection, Method validation

## Abstract

Visual inspection as the golden standard for sleep spindle detection has received mixed support and may not be equally viable across species and experimental conditions. Here we propose a novel intermediate strategy for approximating the quality of a detector and subject our own algorithm for spindle detection in dogs to the procedure. Automatic detections across the full range of our data (excluding only data sets with high subject overlap) were analyzed for their compliance with three face validity criteria, originally discovered by expert scorers in human EEG. These criteria are the shortness of the events (mostly below 2 s, maximum 6), the distribution of faster (> 13 Hz) spindles along the midline (higher in central and posterior derivations) and an association between amplitude and negative chirp (positive for sleep spindles and mostly unrelated for other oscillations). All three criteria were observed in the majority of data sets and sampling events (subsets of data defined by condition and/or recording channel). Replication was more consistent for data sets than for sampling events, but combined probabilities (Fisher’s method) always favoured face validity compliance. Our results indicate that automatic detections in the dog, which were shown in the past to display similar to humans associations with age and cognition, also share face validity criteria with human spindles. This strengthens the dog as a model in sleep spindle research and offers additional arguments for the quality of our detection method.

**Supplementary Information:**

The online version contains supplementary material available at https://doi.org/10.1007/s12021-026-09810-4.

## Introduction

Sleep spindles are a common target in research on sleep-dependent learning and (cognitive) aging (De Gennaro & Ferrara, 2003; Fernandez & Lüthi, 2020; Iotchev & Kubinyi, 2021). They are short-lasting changes in EEG frequency, lasting from half a second (Purcell et al., 2017) to maximum 6 s (Dutertre, 1977), wherein the predominantly slow (maximum 4 Hz) non-REM signal is temporary displaced by a faster rhythm (up to 16 Hz). The best way to quantify sleep spindles is still investigated and debated (Warby et al., 2014).

There are strong arguments to be made both for and against the primacy of visual inspection - once the golden standard. On one hand human scorers, including non-experts, display stronger agreement with each other than was observed for several automated spindle-detectors (Warby et al., 2014). On the other hand the salience and visibility of sleep spindles is not stable across experimental contexts and species. This raises the question if automatically captured spindles that are not confirmed by observers necessarily constitute false positives. It is also possible, that they are instead just consistently below the sensitivity threshold of human scorers (O’Reilly & Nielsen, 2015).

The literature on sleep spindle research in animals (reviewed in Iotchev and Kubinyi (2021) offers good examples for tougher than usual challenges to visual inspection. In the absence-epileptic WAG/Rij rat for example sleep spindles and epileptic spike-wave discharges not only share the same fundamental frequency (van Hese et al., 2009), but intermediate oscillations whose shape is in between that of both phenomena can also be found (Sitnikova et al., 2023) leading to possible misclassifications. Pseudo-spindles of allegedly non-thalamic origin, but similar duration and frequency were observed even in non-epileptic animals (Gottesmann, 1996). Finally, sleep spindles that are more localized can be more easily masked by other frequencies due to volume conduction, and this problem potentially extends to research in humans, as well (O’Reilly & Nielsen, 2015). Among researchers working with non-human EEG, visual inspection is seldom trusted as a means to quantify sleep spindles. Instead, visual scoring was often only used to establish whether spindles are at all present or completely absent (see e.g. Petersen et al. (1964) concerning dogs, cats and rabbits; Pákozdy et al. (2012) concerning dogs, Bert et al. (1970) concerning chimpanzees).

How to reconcile the strengths and weaknesses of both approaches? Although automatic detectors are less likely to miss out on less salient sleep spindles (O’Reilly & Nielsen, 2015), they may still introduce false positives, as well. We here propose a novel type of analysis with which to subject automatic detectors to a quality control. The suggestion is to analyse automatic detections for their compliance with known face-validity indicators that were historically derived through visual inspection. In the present work, we will subject our own automatic detector for sleep spindles in dogs to such an analysis. The detector was first used in Iotchev et al. (2017), and was modelled on the one described by Nonclercq et al. (2013) for use in humans. We will use data sets previously analysed with regard to how sleep spindles in dogs covary with differences in cognition (Iotchev et al., 2020; Bolló et al., 2025), age, and sex (Iotchev et al., 2019). The above mentioned detection method, was consistently used in all these studies, yielding results concurrent with the human literature and thereby indicating high external/predictive validity (Iotchev & Kubinyi, 2021). Face validity was seldom in the focus of this work, with one exception which examined the relative distribution of fast and slow sleep spindles across the scalp midline (Iotchev et al., 2019).

Three indicators of face validity will be used here in particular, because they can be computed from our algorithm’s output without invoking visual inspection (and are, however, derived from visual inspection historically):

1. Sleep spindle duration

Sleep spindles are characteristically short. Although their maximum duration is sometimes given at 6 s (Dutertre, 1977), Purcell et al. (2017) did not observe spindles longer than 2 s in a sample of 11,630 individuals. This not only distinguishes them from alpha waves, which often form long trains (Steriade & Llinás, 1988), but even the shorter pseudo-spindles described by Gottesmann (1996) are on average longer than real thalamic spindles. Schneider et al. (2020) previously reported that their automatic detection for sleep spindles in sheep captures events of short duration, although their algorithm does not specify a maximum duration limit for the search. The authors used this observation as an argument for the validity of their method. A feature to estimate the average duration of our automatic detections was introduced relatively late (ca. 2024) and was of no priority in our previous work where hypotheses related to spindle density (occurrence/min), amplitude and intrinsic frequency (waves/s) were in the main focus. The present work is a first time analysis of the duration of automatically detected dog spindles.

2. Sleep spindle topography

For EEG recordings conducted at the level of the scalp or cortical surface the same pattern is observed consistently in humans (Gibbs & Gibbs, 1964; Jobert et al., 1992; Zeitlhofer et al., 1997), rats (Terrier & Gottesmann, 1978) and dogs (Iotchev et al., 2019). Faster sleep spindles (intrinsic frequency > 13 Hz) occur in larger numbers (higher density) over central and posterior regions, compared to the anterior of the scalp. This relationship between spindle frequency and recording location is confirmed by visual inspection for humans (Gibbs & Gibbs, 1950; Jobert et al., 1992) and rats (Terrier & Gottesmann, 1978). It is the first face validity criterion to be reported for dogs where it concerns automatically detected spindles (Iotchev et al., 2019). Although based on a relatively large sample (*N* > 100) there is currently no reported replication for this observation in the dog. We will analyse this relationship here across all valid data sets (recordings using at least two active electrodes along the midline). Some animal studies report that spindle frequency may also vary as a function of recording depth (Timofeev & Chauvette, 2013; Takeuchi et al., 2016), but this is of no concern here, since all of our data concern non-invasive scalp recordings.

3. Chirp and amplitude

The frequency of a single spindle can change during its short duration, a phenomenon known as ’chirp’. Schönwald et al. (2011)discovered that a negative chirp (decrease in intrinsic frequency between the spindle’s start and end, or deceleration of the spindle’s speed) not only distinguishes spindles from non-spindles, but is related to spindle amplitude (negative chirp was more common in larger spindles). While chirp was automatically quantified in their study, visual inspection was used to verify which events were real spindles. The findings of Schönwald et al. suggest that real spindles should display a positive correlation between amplitude and negative chirp expression, we therefore examine this feature here, as well.

Below we analyse how each of the three face validity criteria appear in our data, how well they replicate across data sets and sampling events (different measurement occasions and/or electrode locations within the same data set) and discuss what this implies for the quality of automatic sleep spindle detection used in the dog so far.

## Methods

### EEG Electrode Montage Used in the Original Studies

**Fig. 1:**
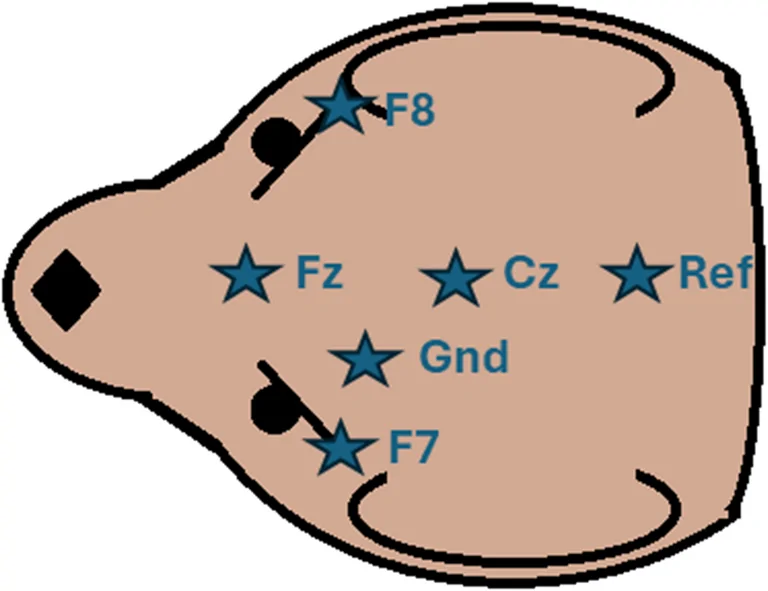
Schematic drawing of the electrode placement in the analysed data sets. Ref = reference, Gnd = ground

All data analysed here was obtained with variations of a single method for non-invasive EEG recording and electrode placement, first described by Kis et al. (2014). In this set-up, all main active electrodes are placed along the skull midline as illustrated in Fig. 1. The occipital bone is the rearmost part of this line and used for placing the general reference. The frontmost placement is the electrode Fz, just above and between the animal’s eyes. With the exception of the earliest studies conducted by our group, the set-up also includes an electrode Cz, placed centrally between Fz and the reference. Three additional electrodes outside the skull midline complete the montage: two channels for monitoring eye movement (F7 and F8) and the ground electrode, placed on the musculus temporalis. A view of the montage from above is demonstrated schematically in Fig. 1.

### Spindle Detection

To ensure that results are comparable across data sets and sampling events we consequently kept using the automatic spindle detection first introduced in Iotchev et al. (2017), so far consistently used in all sleep spindle research on dogs from our group (Iotchev & Kubinyi, 2021). The method is closely modelled on the work of Nonclercq et al. (2013) and follows a two step procedure. First, overlapping time windows of 0.5 s length are evaluated for being part of spindling activity based on an amplitude-frequency criterion (maximum frequency power in the sigma range 9–16 Hz, and root mean square of the time window’s activity required to surpass 1 standard deviation from the average for that recording). Second, estimates of the population mean and standard deviation for amplitude and frequency are derived from the time-windows initially marked as containing sleep spindle activity and events with amplitude and frequency two standard deviations above or below the mean are thrown out in the second selection. Both steps are applied on parts of the signal marked as non-REM sleep by visual inspection.

A function to estimate average spindle duration was added only recently (2024) and was first used in the context of the present work. Sleep spindle duration had not been a priority in our early work and difficult to measure for each spindle separately given our method of detection. Specifically, while the algorithm decides if a time-window contains spindle activity, it cannot differentiate which time-windows are possible starting or end points of the same spindle. For this reason, spindle duration could not be measured at the event-level. We instead derived an estimate by taking into account both the total number of time windows assigned as part of sleep spindle activity, as well as the estimate for separate sleep spindles. Separate spindles were counted if time windows assigned as part of spindling were more than half a second apart. The formula for calculating average duration is given below.

$$\:Average\:duration=\:\frac{N*0.5\:+\:(\:\sum\:w\:-\:N)*0.375}{N}$$

Where N = number of separate sleep spindles, and w = time-windows marked as containing sleep spindle activity (whether or not belonging to separate sleep spindles). The numbers used in this formula refer to the total length of each time-window (0.5 s) and the distance between the centre of two directly neighbouring, overlapping time-windows (0.375).

Another update to our algorithm, specifically for the purpose of this study was a rough estimate for the average negative chirp magnitude. For this purpose we divided the dominant frequency (frequency at which maximum power is observed) left of the center of a spindle-containing time-window by the dominant frequency right of center (for a span of 0.25 s around the center). A higher value obtained by this ratio means that the frequency is decreasing notably as the spindle progresses and this constitutes a negative chirp. The estimate is crude (e.g. we did not use a wavelet-approach) in order to make it align entirely with our existing method such that all chirp estimates correspond in time to the time-windows used in the detection of spindles. It is expected to work on average since the majority of sleep spindles in humans (Purcell et al., 2017), and as we report below, in dogs, are not longer than half a second to a bit over a second maximum.

### Data and Observations

All data consisted of polysomnographic recordings from afternoon naps in domestic dogs, with a duration of 2 to 3 h/recording. The compliance of automatic detections with face-validity criteria was tested within and across sampling events, but also where applicable entire data sets. A sampling event is defined here as a unique combination of sampled population, experimental condition, and electrode from which the physiological data was collected (Fz or Cz). In this way the re-test reliability of the compliance was tested not only across different sets of subjects, but also across different conditions for the same subjects. Looking at sampling events separately allowed us to test if certain observations were more likely to repeat under certain conditions (e.g. during adaptation recordings or for spindles recorded from the same electrode). We thus primarily report the effect sizes and p-values for each sampling event separately, but in the discussion we elaborate on the extent to which replication was observed across populations versus different tests on the same subjects. With the exception of one dog (female golden retriever, between 1 and 2 years old at the time of data collection), there was no overlap between data sets with regard to sampled population. If data sets presented with substantial overlap in subjects only the larger data set was included in the meta analysis. In total we looked at 20 sampling events from 5 data sets, with the aforementioned subject appearing in 3 of the 5 data sets. The sampling events, data sets and valid sample size (valid N) are reported below in Table 1.

**Table 1:** Each combination of data set, condition, and electrode (referred to later on as sampling event) with the corresponding valid sample size (N). Experimental conditions are referenced through abbreviations for some of the studies, i.e. “sup” = supportive, “con” = controlling (referencing two different strategies for training); “rel” = (socially) relevant, “irrel” = (socially) irrelevant (referencing two types of demonstration during a learning experiment); finally “exp” refers to the experimental condition in the TMR study for which there was only one experimental condition and hence different codes for different conditions were not required here

Data set	Condition	Electrode	Valid *N*
CL0^*^	control	Fz	15
CL0	learning	Fz	15
CL1	adaptation	Fz	14
CL1	adaptation	Cz	14
CL1	sup	Fz	17
CL1	sup	Cz	17
CL1	con	Fz	19
CL1	con	Cz	17
CL2	adaptation	Fz	11
CL2	adaptation	Cz	11
CL2	rel	Fz	13
CL2	rel	Cz	13
CL2	irrel	Fz	12
CL2	irrel	Cz	12
ASX	adaptation	Fz	146
ASX	adaptation	Cz	85
TMR	adaptation	Fz	16
TMR	adaptation	Cz	16
TMR	exp	Fz	16
TMR	exp	Cz	16

^*^No adaptation recordings are listed here for data set CL0, since these were originally analyzed separately as part of data set ASX, while the adaptation recordings from data sets CL1, CL2 and TMR were obtained later and analyzed separately from the ones analyzed in the ASX data set

The data sets are referenced by initials referring to the original study purpose. CL0 to CL2 are data sets used to study the relationship between sleep spindle occurrence and Command Learning (hence CL), these three were analysed together also in Iotchev, Reicher, Kovács et al. (2020), but there with a focus on predicting cognition. ASX (Age and SeX) was a large (*N* > 100) sub-sample of our continuously growing data base for polysomnographic adaptation recordings in dogs, used to analyse how the animals’ sleep spindles vary with age, sex and reproductive status (Iotchev et al., 2019). Finally, TMR (Targeted Memory Reactivation) was a data set collected to analyse if targeted memory reactivation works in dogs (Bolló et al., 2025). Data sets were previously shared either as stand-alone data repository entries (see https://osf.io/96hj7/overview for TMR) or as supplementary data within the articles (see https://www.nature.com/articles/s41598-017-13278-3#Sec14 for CL0; https://www.nature.com/articles/s41598-020-80417-8*for CL1 and CL2*; https://www.nature.com/articles/s41598-019-46434-y for ASX). Variables specifically calculated for the analyses conducted here are shared in this article’s supplementary, while the most up to date version of our detection algorithm for sleep spindles can be found on GitHub (https://github.com/IvoWolf1987/spindle_hound).

### Statistical Analyses

For each sampling event we used (depending on the question) paired t-tests, simple Pearson correlations or even descriptive statistics (for the duration of automatically detected spindles). When an entire data set was analysed, a Generalized Linear Mixed Model (GLMM) was used to account for the fact that each subject yielded several data points (due to two active electrodes: Fz and Cz, and usually 2 to 3 experimental conditions). Since most of our variables are strictly positive, a Gamma distributions assumption was used for the GLMMs (Tweedie if zeros were present). We also included subject, condition, and/or electrode as random factors. Simpler analyses were performed in SPSS v25, whereas for GLMMs we implemented glmmTMB (Brooks et al., 2017; McGillycuddy et al., 2025) in R (R Development Core Team, 2015) using the following syntax:

$$\:model\:<-\:glmmTMB\left(\begin{array}{c}y\sim\;x\:+\:\left(1\vert R1\right)\:+\:\left(1\right\vert R2),\:family\;=\\\;Gamma/tweedie(link\:=\;^{\prime\prime} log^{\prime\prime})\end{array}\right)$$

Where y = dependent variable, x = independent variable and Rn = nth random factor. The exact identity of the random factors depended on the hypothesis, e.g. when testing differences between electrodes, electrode was not included as a random factor, but it was a random factor when testing for associations between chirp and amplitude.

Additional combined probabilities (across data sets and/or sampling events) were calculated, likewise in R using Fisher’s method (Fisher, 1970), using the function *sumlog* (Dewey, 2019).

## Results

### Average Duration of Spindle Detections

Table 2

**Table 2:** Means (across the entire sample), standard deviations and ranges of averaged (across a recording) spindle durations (in seconds)

Data set	Condition	Electrode	Mean (seconds)	SD	Range
CL0	control	Fz	0.79	1	0.51–4.25
CL0	learning	Fz	0.54	0.02	0.50–0.58
CL1	adaptation	Fz	0.55	0.08	0.50–0.75
CL1	adaptation	Cz	0.55	0.06	0.50–0.67
CL1	sup	Fz	0.56	0.07	0.50–0.69
CL1	sup	Cz	0.56	0.07	0.50–0.79
CL1	con	Fz	0.52	0.02	0.50–0.56
CL1	con	Cz	0.54	0.04	0.50–0.62
CL2	adaptation	Fz	0.56	0.05	0.5–0.7
CL2	adaptation	Cz	0.58	0.05	0.53–0.68
CL2	rel	Fz	0.55	0.02	0.52–0.59
CL2	rel	Cz	0.59	0.06	0.53–0.74
CL2	irrel	Fz	0.54	0.02	0.51–0.57
CL2	irrel	Cz	0.57	0.04	0.51–0.70
ASX	adaptation	Fz	0.56	0.08	0.50–1.30
ASX	adaptation	Cz	0.60	0.09	0.50–1.06
TMR	adaptation	Fz	0.60	0.10	0.50–0.88
TMR	adaptation	Cz	0.61	0.14	0.50–1.09
TMR	exp	Fz	0.60	0.06	0.53–0.74
TMR	exp	Cz	0.65	0.14	0.52–1.08

### Topography of Fast and Slow Sleep Spindles

The combined probability for comparing fast spindle density between Fz and Cz was significant for analysing across data sets (ꭕ^2^ = 104.71, df = 8, *P* < 0.001) and also across sampling events (ꭕ^2^ = 70.70, df = 18, *P* < 0.001). All significant effects were of the same direction (Cz > Fz). The individual p-values are displayed in Table 3 (for data sets) and Table 4 (for sampling events).

**Table 3:** Effect sizes, confidence intervals, and p-values for fast spindle density on Fz versus Cz across all data sets

data set	β	95% CI	*p*-value	effect direction
**CL1**	**1.166**	**0.725–1.608**	**< 0.001**	**Cz > Fz**
**CL2**	**0.940**	**0.504–1.375**	**< 0.001**	**Cz > Fz**
**ASX**	**0.851**	**0.581–1.120**	**< 0.001**	**Cz > Fz**
**TMR**	**0.717**	**0.210–1.224**	**0.006**	**Cz > Fz**

**Table 4:** Paired t-test t-values and p-values for fast spindle density on Fz versus Cz across all sampling events

Data set	Condition	Effect size (t)	*p*-value	Effect direction
CL1	adaptation	1.381	0.191	Cz > Fz
**CL1**	**sup**	**2.808**	**0.013**	**Cz > Fz**
**CL1**	**con**	**2.702**	**0.016**	**Cz > Fz**
**CL2**	**adaptation**	**2.696**	**0.022**	**Cz > Fz**
CL2	rel	1.905	0.081	Cz > Fz
CL2	irrel	2.115	0.058	Cz > Fz
**ASX**	**adaptation**	**3.966**	**< 0.001**	**Cz > Fz**
TMR	adaptation	1.708	0.108	Cz > Fz
**TMR**	**exp**	**4.536**	**< 0.001**	**Cz > Fz**

The combined probability for comparing slow spindle density between Fz and Cz was not significant for analysing across data sets (*P* = 0.225), nor across sampling events (*P* = 0.052). Although the combined probabilities are trends, the direction of the effect was not the same for all significant tests. The individual p-values are displayed in Table 5 (for data sets) and Table 6 (for sampling events).

**Table 5:** Effect sizes, confidence intervals, and p-values for slow spindle density on Fz versus Cz across all data sets

Data set	β	95% CI	*p*-value	Effect direction
CL1	0.003	-0.299–0.306	0.984	Cz < Fz
**CL2**	**0.229**	**0.032–0.426**	**0.023**	**Cz < Fz**
ASX	0.073	-0.065–0.211	0.299	Cz < Fz
TMR	0.070	-0.334–0.474	0.735	Cz < Fz

**Table 6:** Paired t-test t-values and p-values for slow spindle density on Fz versus Cz

Data set	Condition	Effect size (t)	*p*-value	Effect direction
CL1	adaptation	0.454	0.657	Cz < Fz
CL1	sup	0.498	0.625	Cz > Fz
CL1	con	0.602	0.556	Cz > Fz
CL2	adaptation	1.259	0.237	Cz < Fz
CL2	rel	1.797	0.098	Cz < Fz
CL2	irrel	1.243	0.240	Cz < Fz
**ASX**	**adaptation**	**2.210**	**0.030**	**Cz < Fz**
**TMR**	**adaptation**	**2.346**	**0.033**	**Cz > Fz**
TMR	exp	0.760	0.459	Cz < Fz

### Amplitude and Negative Chirp

In Table 7 we list the effect-sizes (F) and p-values for each of the five data sets (using GLMM to account for which observations come from the same subjects), while in Table 8 we list the effect-sizes (r) and p-values for each sampling event (i.e. each combination of data set, condition and electrode). Results in each table concern the correlation between average spindle amplitude and average negative chirp magnitude. The combined probability was significant across data sets (ꭕ^2^ = 69.12, df = 10, *P* < 0.001) and sampling events (ꭕ^2^ = 122.74, df = 40, *P* < 0.001). All significant findings concern the same direction for the effect - a positive correlation between average amplitude and average negative chirp magnitude.

**Table 7:** Effect-sizes, 95% confidence intervals, and p-values for the association between average spindle amplitude and average negative chirp magnitude, analysed for each data set

Data set	β	95% CI	*p*-value
CL0	-0.461	-1.730216e + 00–8.082019e-01	0.477
**CL1**	**0.450**	**0.122–0.779**	**0.007**
**CL2**	**0.745**	**0.394–1.097**	**< 0.001**
**ASX**	**0.575**	**0.282–0.868**	**< 0.001**
**TMR**	**1.334**	**0.673–1.994**	**< 0.001**

**Table 8:** Shown are r and p-values for the association between average spindle amplitude and average negative chirp magnitude, analysed for each sampling event

Data set	Condition	Electrode	*R*	*p*-value
CL0	control	Fz	− 0.266	0.338
CL0	learning	Fz	0.313	0.256
**CL1**	**adaptation**	**Fz**	**0.760**	**0.002**
**CL1**	**adaptation**	**Cz**	**0.810**	**0.001**
**CL1**	**sup**	**Fz**	**0.767**	**< 0.001**
CL1	sup	Cz	0.319	0.211
CL1	con	Fz	− 0.023	0.924
**CL1**	**con**	**Cz**	**0.618**	**0.008**
CL2	adaptation	Fz	0.095	0.782
**CL2**	**adaptation**	**Cz**	**0.722**	**0.012**
CL2	rel	Fz	− 0.223	0.464
CL2	rel	Cz	0.143	0.640
**CL2**	**irrel**	**Fz**	**0.579**	**0.049**
**CL2**	**irrel**	**Cz**	**0.613**	**0.034**
ASX	adaptation	Fz	0.077	0.359
**ASX**	**adaptation**	**Cz**	**0.289**	**0.007**
TMR	adaptation	Fz	0.493	0.052
TMR	adaptation	Cz	− 0.135	0.617
**TMR**	**exp**	**Fz**	**0.859**	**< 0.001**
**TMR**	**exp**	**Cz**	**0.567**	**0.022**

## Discussion

Previous work on dog sleep spindles primarily focused on external validity, i.e. how certain experimental effects like e.g. exposure to learning prior sleep replicate across species, comparing sleep spindles in the dog with what is known from humans and rodents (reviewed in Iotchev and Kubinyi (2021). Here we expand on the face validity of automatically detected dog spindles from the same data sets for which external validity is well reported, and for which the same automatic detection was consistently used.

**Fig. 2:**
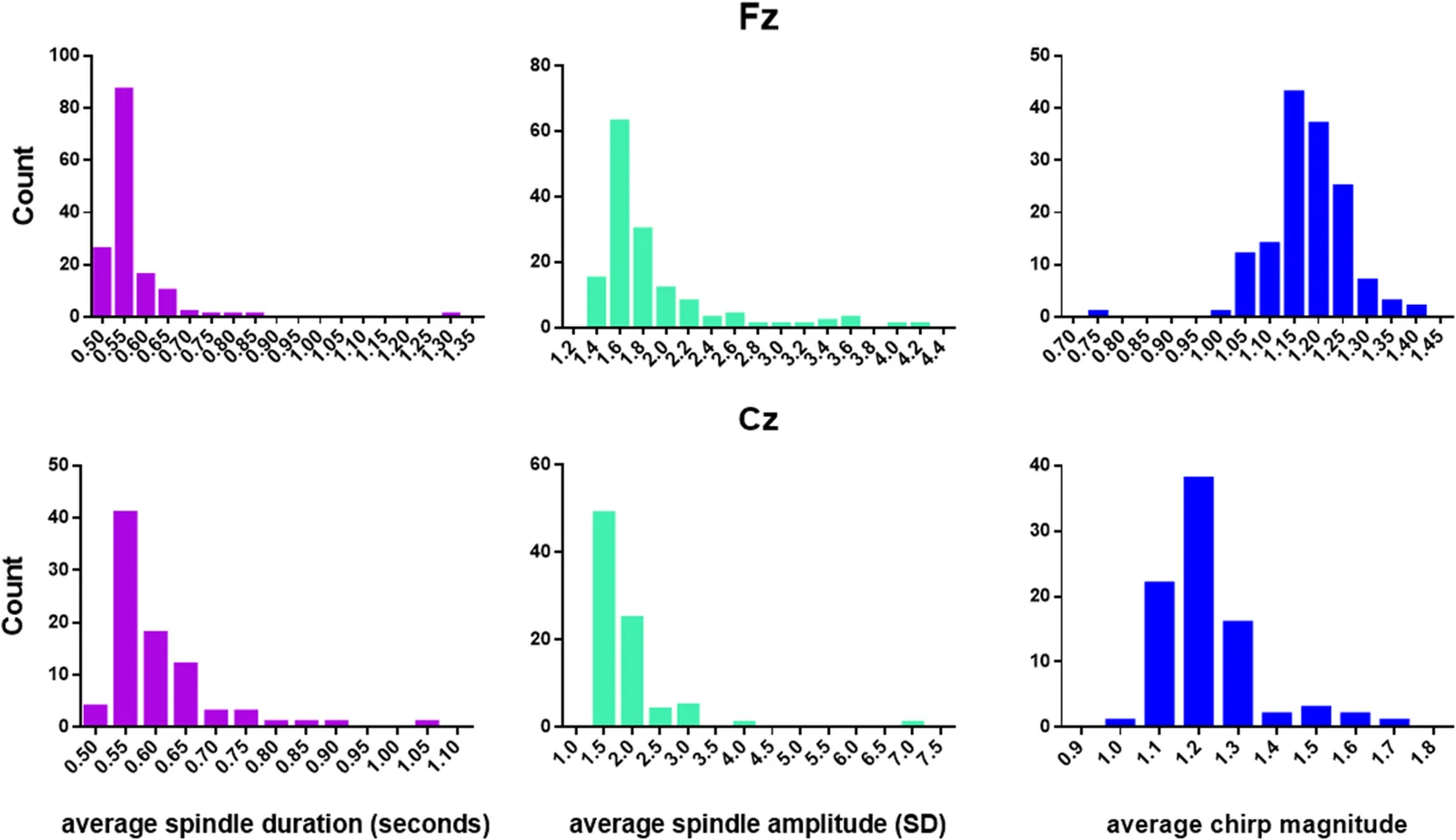
Histograms for the distribution of average duration, amplitude and (negative) chirp magnitude in our largest data set (ASX), displaying the number of subjects for each value

Overall, we report that three features of spindles that were originally observed under visual inspection in humans and other animals also replicate in automatically detected spindles from the dog. The distribution of these features are visually summarized in Fig. 2 for our largest data set (ASX) and are discussed in detail below.

### Duration

Across all 20 sampling events from our 5 data sets we only once observe a recording from a dog where the average spindle duration would exceed one and half seconds. Even the longest average spindle duration for a recording in our data sets is below the 6 s maximum reported in the literature (Dutertre, 1977). The average durations for a sampling event (pooled across the averages of each recording) are consistently below a second, while the variation is also consistently low (SD ≤ 1, and for all but one sampling event SD < 0.2). We can exclude that the possible average duration estimates are limited by the size of our shifting time-window, since average durations exceeding the length of this window (i.e. > 0.5 s) were common. Although some authors have contended that spindles can last up to 6 s (Dutertre, 1977), the vast majority, across thousands of study subjects (*N* = 11630) are characteristically much shorter, most falling within a range from half a second to maximum 2 s (Purcell et al., 2017). Shortness is an important face validity criterion. It not only distinguishes sleep spindles from the much longer, although otherwise similar trains of alpha activity (Steriade & Llinás, 1988), but also from the relatively short (but on average still longer) pseudo-spindles observed by Gottesmann (1996). An actual, direct comparison with the latter, however is not possible at this point. For this we will need to develop a function with which to measure duration spindle by spindle, and also a method for identifying Gottesmann’s pseudo-spindles in the dog. We are the second group to our knowledge, which found that a realistic for spindles duration is observed for a detection method that does not apply an upper limit on duration during the automated search. This observation was previously used to validate an automatic detection for sleep spindles in sheep (Schneider et al., 2020). Our method, however, also includes a limited search to parts of the signal marked as non-REM sleep, where long alpha-trains are not common. Whether this sufficiently explains the compliance with short duration remains to be examined in future work. Here we focused on the detections that were made in previous studies, so that our previous work on external/predictive validity can be compared and complemented by an analysis of face validity criteria.

### Topography

Recordings at the level of the cortical surface are expected to show a different distribution for slow (< 13 Hz) and fast (> 13 Hz) spindles along the midline. In humans and rats fast spindles are more abundant in central and posterior parts of the cortex, while slow spindles predominate over the anterior lobe (Gibbs & Gibbs, 1950; Terrier & Gottesmann, 1978; Jobert et al., 1992; Zeitlhofer et al., 1997). In dogs we find ample support for a similar topography when the distribution of fast spindles is compared between the frontally placed Fz and the central Cz electrode (the main active electrodes in our set-up, see also (Kis et al., 2014; Iotchev et al., 2023). Specifically, we observe that a higher density for fast spindles is consistently found at Cz. This contrast was significant in all four data sets (only four out of five data sets were suitable for this analysis, since no active Cz electrode had been used in CL0). Looking at separate sampling events, this difference was at least a trend (*P* < 0.1) for 7 of 9 sampling events (77.8% of sampling events) and statistically significant (at alpha = 0.05) for 5 of 9 sampling events (or 55.6%), including the sampling event corresponding to our largest data set (*N* = 146, but valid *N* = 85 since not all animals presented data from both electrodes). Finally, the combined probabilities (Fisher’s method) for data sets and sampling events were likewise significant for this comparison. Differences in slow spindle density between Fz and Cz were less reliable than the ones observed for fast spindles. The comparisons were mostly not significant, and showed variation in the direction of the effect. This discrepancy might reflect how electrode placement aligns with the sources of slow and fast spindles. At least in rats (Terrier & Gottesmann, 1978) fast spindles may originate as far back as the occipital cortex. Assuming a similar localization in the dog would mean that our Cz electrode is closer to the source of slow spindles (in all mammals that is the frontal cortex) than Fz is to the source of fast spindles. Our electrode placement therefore likely amplifies localization differences in fast spindle occurrence, but not for slow spindle occurrence.

### Amplitude and Negative Chirp

Schönwald et al. (2011) observed that for visually confirmed sleep spindles in humans, amplitude and negative chirp were correlated, i.e. larger spindles displayed a deceleration (a drop in intrinsic frequency) between start and end. Here we used a relatively crude method for quantifying negative chirp in order to make chirp estimates align precisely with the estimated (by our algorithm) time points for sleep spindle activity. Our chirp estimation works best (most accurately) under the assumption of short (maximum second long) spindles that are detected at the center. This assumption is satisfied for most sleep spindles in humans (Purcell et al., 2017), but also, as we have shown here, for dogs (see results and discussion of average duration values, as well as Fig. 2). We observed a correlation between amplitude and negative chirp magnitude (values represent averages/recording) in four out of five data sets. This included our largest data set (*N* = 146). The combined probabilities for data sets and sampling events were likewise significant, but looking at sampling events separately the effect was more reliably replicated for sleep spindles detected on Cz (significant at alpha = 0.05 for 6 out of 9 events sampled at Cz). This is interestingly not the first time that humanlike sleep spindle dynamics in the dog are observed specifically for spindles detected on Cz. In our previous work, age-related decline in sleep spindle occurrence and increase in sleep spindle intrinsic frequency were also observed only over Cz (Iotchev et al., 2019). This raises questions about the quality of Cz versus Fz signals with regard to sleep spindle detection and analysis. Sleep spindles as correlates of sleep-dependent learning seem exclusive to Fz in the dog, but are also better detected when estimates of sleep spindle numbers are averaged across available recordings (Iotchev et al., 2017, 2020). This suggests an interaction between our method, the electrode and the actual source of the effect. Fz may be the overall noisier channel, but still the preferred choice for quantifying anterior sleep spindles.

### General Discussion and Conclusions

We analysed 20 sampling events from five data sets to evaluate how automatically detected sleep spindles in the dog, obtained consistently with the same algorithm, comply with known face validity criteria. We observe that three features, initially confirmed with the aid of visual inspection to characterize human sleep spindles, are replicated in the majority of data sets and/or sampling events analysed in the dog: short duration (Purcell et al., 2017), the concentration of fast (> 13 Hz) spindles along the midline (Gibbs & Gibbs, 1950; Jobert et al., 1992; Zeitlhofer et al., 1997), and a more pronounced negative chirp in larger spindles (Schönwald et al., 2011). These observations add additional support to both the face validity of dogs’ sleep spindles (as a model for human sleep spindles) but also for the quality of the detection method used consistently throughout our studies on the canine spindle.

Possible other features to examine in the future or add as a quality control for automatic detectors concerns the tendency of sleep spindles to form clusters of increased incidence with short inter-spindle intervals (Steriade & Llinás, 1988; Antony et al., 2018; Chen et al., 2025). The here examined detection method does not provide a good basis for this type of analysis, because it does not distinguish which time-windows occupied by spindle activity form starting or end points of individual spindles. For this very same reason measuring average duration and chirp required crude work-arounds which were not conceived during earlier versions of our detector.

Face validity compliance suggests that the algorithm has reasonable specificity (selectivity). A too inclusive detection with too many false positives in its final selection would deviate randomly from complying with face validity, but we instead observe a replication for all three criteria across the examined data sets. A more precise estimate for the specificity of a detector is usually achieved by a comparison with expert scorers, but for sleep spindles this approach has been questioned (O’Reilly & Nielsen, 2015), and specifically in animal research using visual inspection to estimate sleep spindle numbers has been avoided (Petersen et al., 1964; Bert et al., 1970; Pákozdy et al., 2012). The here offered argument for the specificity of our algorithm adds up with previous findings supporting its sensitivity by demonstrating cross-species replications for known effects of age, sex, and learning on the expression of sleep spindles (Iotchev et al., 2017, 2019, 2020; Bolló et al., 2025).

We were also able to detail how sample size and recording site (Fz versus Cz) affect the visibility of the reported effects. Replication was more consistent across data sets than across the subsets we referred to as ’sampling events’. This is in line with the expectation that type II statistical errors will be more common in smaller samples (Schmidt & Hunter, 2004). In spite of their lower statistical power, the subsets of data defined by condition and/or electrode were still mostly in agreement with the better powered, undivided data sets and the combined probabilities, calculated with Fisher’s method (Fisher, 1970), were also significant. Although at times by a small margin, effects in line with the literature were the majority across sampling events. Importantly, the results obtained for different subsets of data give us insight into the likelihood of observing correct results in small (*N* ≤ 16) samples, using the agreement between the literature and the larger, undivided data sets as a reference point for what is correct. The likelihood for true effects to show up in small samples is an important issue, since sample sizes of *N* < 20 are extremely common in the literature on sleep spindles (Gais et al., 2002; Clemens et al., 2005, 2006; Cox et al., 2012; Iotchev et al., 2017, 2020).

Given that some authors have produced valuable arguments in favour of using visual inspection (Warby et al., 2014) we do not discourage referencing against expert scorers per sé. However, the here proposed analysis protocol allows to approximate the quality of automatic detection when the possibility for visual inspection is limited by noisy data (e.g. the presence of spindle-like distractors in epileptic animals (van Hese et al., 2009; Sitnikova et al., 2023) or when visual inspection is highly impractical. In our case, for example, the here reported analyses allowed us to time-efficiently estimate detection quality across the almost complete range of our data (excluding only data sets with high subject overlap). To use visual inspection as a reference point time-efficiently would have required work with smaller subsamples which inevitably increases the chance of sampling bias. Finally, the approach used here is an integrated response to the arguments offered for and against visual inspection (Warby et al., 2012; O’Reilly & Nielsen, 2015).

## Supplementary Information

Below is the link to the electronic supplementary material.

Supplementary Material 1

## Data Availability

Values calculated specifically for these analyses are shared in a Supplementary collection of excel tables. The original data had also been shared before alongside several published works.
